# Roseomonas Species Bacteremia With Associated Endocarditis and Possible CNS Septic Embolic Phenomenon

**DOI:** 10.7759/cureus.40318

**Published:** 2023-06-12

**Authors:** Jose A Rodriguez, Alexis C Gushiken

**Affiliations:** 1 Infectious Diseases, University of Florida, Gainesville, USA; 2 Infectious Disease, University of Florida, Gainesville, USA

**Keywords:** drug susceptibility testing and antibiotic resistance, septic emboli, bacteremia, endocarditis, roseomonas

## Abstract

*Rosemonas* species has been associated with infections in both immunocompetent and immunocompromised hosts, manifesting as peritonitis, bacteremia, catheter-related bacteremia, endophthalmitis, spondylitis, and endocarditis. Here we present a man in his 60s who was brought to our institution for sudden onset of aphasia, right-sided paresthesia, and new onset tonic-clonic seizure episodes. At presentation, he was found to have severe lactic acidosis, acute kidney failure, bilateral hydronephrosis, elevated prostate-specific antigen (PSA), and an enlarged prostate. Blood cultures obtained on admission later grew *Roseomonas* species for which he was started on meropenem. A trans-esophageal echocardiogram (TEE) showed multiple very thin mobile densities on the ventricular side of the aortic valve; magnetic resonance imaging (MRI) of the brain revealed an 11 mm acute/subacute hemorrhage. The patient was discharged in stable condition on Ertapenem intravenous therapy for six weeks. *Roseomonas mucosa* can be a cause of endocarditis. The antimicrobial resistance profile of *Roseomonas spp* suggests that carbapenems, fluoroquinolones or aminoglycosides are the drugs of choice for Roseomonas infections and that infectious diseases involved in cases of *Roseomonas* infections should be instituted promptly for proper management.

## Introduction

*Rosemonas mucosa* has been associated with infection in different hosts immunocompetent and immunocompromised manifesting as peritonitis, bacteremia, catheter-related bacteremia, endophthalmitis, spondylitis [1-6], one case by Shao et al. reported a case of infective endocarditis in a patient with lupus [7]. Its resistance to cephalosporins including cefepime makes it a health problem that needs to be identified promptly to prevent the development of complications related to infection. Here we present a man in his 60s with successfully treated *Roseomonas mucosa *bacteremia causing endocarditis and possibly CNS septic emboli.

## Case presentation

A 63-year-old male with no significant past medical history was brought to the emergency department after developing aphasia, dizziness, and right-sided paresthesia. He was last seen to be normal one hour prior to arrival. En route to the hospital, the patient had a witnessed tonic-clonic seizure event and was found to be in a post-ictal state in the emergency department with a Glasgow coma scale of 3. His blood pressure was 231/135 mmHg, heart rate 135 beats per minute, and temperature 37.1 degrees Celsius. He underwent emergent computed tomography (CT) angiogram of the head with no perfusion abnormality identified. A CT abdomen and pelvis showed marked thickening and irregularity of the bladder wall and an enlarged prostate gland. Laboratory workup was significant for white blood cell (WBC) 12.9 thou/mm^3^, lactic acid 10.9 mmol/L, high-sensitivity CRP 30.61 mg/L, creatinine 7.3 mg/dL, prostate-specific antigen (PSA) 28 ng/ml.

One set of aerobic and anaerobic blood cultures was obtained in the emergency department, and the patient was started on empiric piperacillin/tazobactam 3.375 gm intravenous (IV) every eight hours for sepsis. The patient had an indwelling urinary catheter placed for urinary retention. A urinalysis was sent that revealed 4 WBC per high power field (HPF), 150 RBC per HPF, negative nitrite, and negative leukocyte esterase. The urinalysis results did not meet the criteria to reflex to culture.

The patient had a subsequent episode of myoclonic seizure and was intubated for airway protection. A lumbar puncture was performed with cerebrospinal fluid (CSF) analysis revealing protein 58 mg/dL, glucose 69 mg/dL, WBC 1, and no organisms on gram stain or cultures. CSF Biofire FilmArray ME polymerase chain reaction (PCR) test had no pathogens detected. Piperacillin/tazobactam was discontinued due to the low suspicion of bacterial meningitis based on the CSF findings.

On hospital day 4, the initial blood cultures drawn in the emergency department grew *Roseomonas* species in the aerobic bottle. The organism was identified as *Roseomonas gilardii* by the Vitek2 system (bioMerieux, Durham, NC, USA) and *Roseomonas mucosa* by matrix-assisted laser desorption ionization time-of-flight mass spectrometry (MALDI-TOF MS) (bioMerieux, Durham, NC, USA). Antibiotic susceptibility testing was obtained using ETEST® (bioMerieux, Durham, NC, USA), demonstrating resistance to ceftazidime, piperacillin-tazobactam, and aztreonam, as shown in Table 1.

**Table 1:** Roseomonas species susceptibility tested with ETEST® MIC/ETEST: ETEST® (bioMerieux, Durham, NC, USA) is a well-established method for Minimum Inhibitory Concentration (MIC) determinations in microbiological investigations. The antibiotics with E Test written next to them show that the MIC determination tests were done manually.

Antibiotics	MIC/ETEST	Susceptibility
Amikacin E Test	0.75 ug/mL	Susceptible
Aztreonam	32 ug/mL	Resistant
Cefepime	6 ug/mL	Susceptible
Ceftazidime E Test	256 ug/mL	Resistant
Ceftriaxone E Test	2 ug/mL	Susceptible
Ciprofloxacin E Test	0.25 ug/mL	Susceptible
Colistin E Test	6 ug/mL	Susceptible
Ertapenem	0.19 ug/mL	Susceptible
Gentamicin E Test	0.25 ug/mL	Susceptible
Levofloxacin E Test	0.19 ug/mL	Susceptible
Meropenem E Test	0.19 ug/mL	Susceptible
Minocycline E Test	0.094 ug/mL	Susceptible
Piperacillin/Tazobactam E Test	256 ug/mL	Resistant
Tobramycin E Test	0.094 ug/mL	Susceptible
Trimethoprim/Sulfa E Test	0.75 ug/mL	Susceptible

The Infectious Disease service was consulted for management. Repeat blood cultures were drawn and the patient was started on meropenem 1 gram IV every eight hours. The repeat blood cultures were ultimately negative. To evaluate for a source of the seizures, brain magnetic resonance imaging (MRI) was performed and showed an 11 mm acute/subacute hemorrhage with vasogenic edema in the left middle frontal gyrus at the level of premotor gyrus, and a 3.4 mm area of abnormal contrast enhancement in the central sulcus on the right. These findings were concerning for a possible embolic stroke.

**Figure 1:**
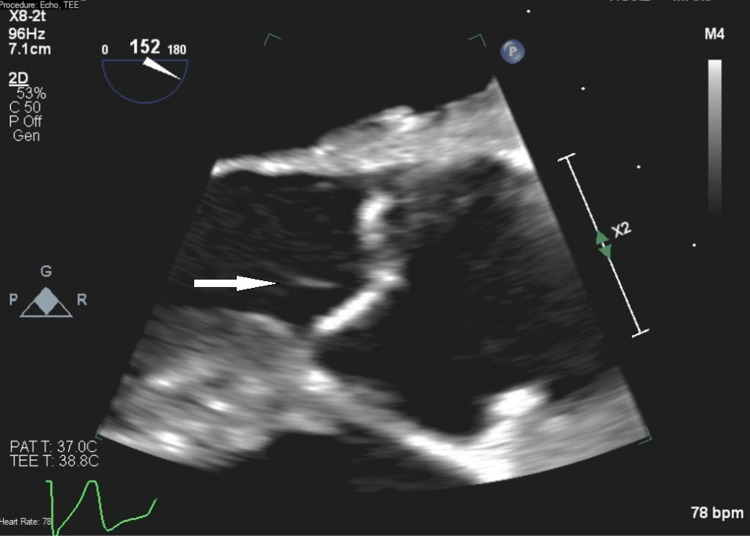
Trans-esophageal echocardiogram The white arrow denotes a very thin mobile density seen on the ventricular side of the aortic valve

A trans-thoracic echocardiogram (TTE) showed no evidence of valvular vegetations. A trans-esophageal echocardiogram (TEE) (Figure 1) revealed multiple very thin mobile densities seen on the ventricular side of the aortic valve (as long as 8 mm). Due to the lack of valvular dysfunction and the small size of the vegetation, no surgical intervention was pursued.

The patient has transitioned to ertapenem 1 gm daily IV. Urology was consulted inpatient for cystoscopy and prostate biopsy, which were deferred for outpatient follow-up. Prostate biopsy was eventually obtained with pathology notable for adenocarcinoma. The patient remained afebrile thorough admission, his aphasia resolved, and he had no more episodes of seizure events noted. He was discharged home to complete a six-week course of therapy with IV Ertapenem. He followed up with an infectious disease outpatient upon completion of IV therapy and felt well with no residual neurologic deficits. He also followed up with urology for a prostate biopsy which resulted positive for adenocarcinoma of the prostate. He later underwent photovaporization of the prostate.

## Discussion

*Roseomonas mucosa* is a slow-growing, non-endospore forming, pink-pigmented gram-negative coccobacillus. It belongs to the genus *Roseomonas *which was first described by Rihs et al. in 1993 [8]. Some *Roseomonas* isolates have been recovered from environmental water sources, but the majority were isolated from blood, CSF, wounds, eyes, and bone [8]. In our case, the suspected primary source of infection is a urinary tract infection secondary to urinary retention related to newly-diagnosed prostate cancer. A study done by Han et al. characterized 36 strains of *Roseomonas* species isolated from blood. All strains were susceptible to amikacin and ciprofloxacin; resistant to cefepime, ceftazidime, imipenem, and ticarcillin-clavulanate; and overall less susceptible to ceftriaxone, trimetroprim-sulfamethoxazole, and ampicillin [9].

The patient was found to be bacteremic with *Roseomonas* species, which is an uncommon pathogen with known resistance to many antibiotics. Workup for infective endocarditis was pursued given the potential embolic stroke in the setting of bacteremia. The TTE did not show evidence of infective endocarditis, but the subsequent TEE revealed mobile vegetations concerning endocarditis. These types of thin densities can be seen with degenerative changes of the aortic valve (called Lambl's excrescences), but the presence of bacteremia and location of the densities on the ventricular side of the leaflet highly suggest infective vegetations. 

In our case, a decision to start meropenem was made due to superior CNS penetration, given the concern for a septic embolism as the source of his hemorrhagic stroke. We transitioned to ertapenem based on susceptibilities and for ease of administration while on home IV therapy. The six-week treatment duration was established to cover treatment for native valve infective endocarditis as per American Heart Association and Infectious Disease Society of America guidelines [10]. A case report by Shao et al. described a patient with *Roseomonas mucosa* infective endocarditis in a patient with lupus erythematosus [7]. Similar to our case, they reported that *Roseomonas* was identified by the Vitek2 system as *Roseomonas gilardii* and the MALDI-TOF MS identified *Roseomonas mucosa;* they confirmed the identity of the isolate as *Roseomonas mucosa* using a fragment of the 16S rRNA PCR amplification [7]. A comparison study done by Rudolph et al. compared Vitek2, MALDI-TOF MS 16S rRNA gene sequencing for the identification of *Roseomonas mucosa*; they concluded that MALDI-TOF MS and 16S rRNA gene sequencing confidently identified the species however using Vitek2 isolates were misidentified [11].

## Conclusions

*Roseomonas mucosa* can be a cause of endocarditis. The antimicrobial resistance profile of *Roseomonas spp* suggests that carbapenems, fluoroquinolones, or aminoglycosides are the drugs of choice for *Roseomonas* infections and that infectious diseases involvement in cases of *Roseomonas* infections should be instituted promptly for proper management. Also, it is notable that MALDI-TOF MS is more specific at identifying *Roseomonas spp* compared to Vitek2.
